# Supplementary material for: Comparing Multi-Walled Carbon Nanotubes and Halloysite Nanotubes as Reinforcements in EVA Nanocomposites
Source: Materials (Basel). 2020 Aug 28;13(17):3809. doi: 10.3390/ma13173809 (PMC7504041; doi:10.3390/ma13173809)
Supplement: Supplementary file 1 [file materials-13-03809-s001.zip › materials-888502-supplementary.docx]

Supplementary Materials

Comparing Multi-Walled Carbon Nanotubes and Halloysite Nanotubes as Reinforcements in EVA Nanocomposites

Agata Zubkiewicz ^1,^*, Anna Szymczyk ^1^, Piotr Franciszczak ^2^, Agnieszka Kochmanska ^2^, Izabela Janowska ^3^ and Sandra Paszkiewicz ^2,^*

^1^ Department of Technical Physics, West Pomeranian University of Technology, 48, 70311 Szczecin, Poland; aszymczyk@zut.edu.pl

^2^ Department of Materials Technology, West Pomeranian University of Technology, 19, 70310 Szczecin, Poland; piotr.franciszczak@zut.edu.pl (P.F.); akochmanska@zut.edu.pl (A.K.)

^3^ Institut de Chimie et Procédés pour l’Energie l’Environnement et la Santé (ICPEES), University of Strasbourg, 67087, France; janowskai@unistra.fr

***** Correspondence: agata.zubkiewicz@zut.edu.pl (A.Z.); spaszkiewicz@zut.edu.pl (S.P.); Tel.: +48-91-449-4589

| 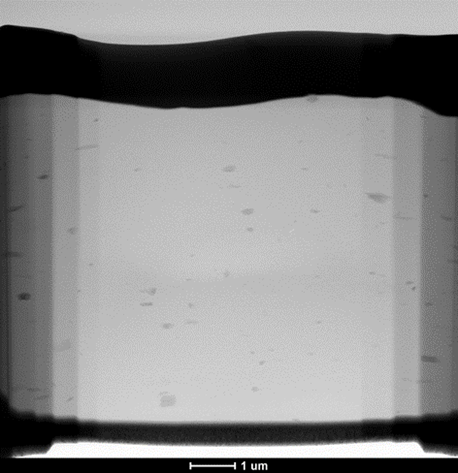 | 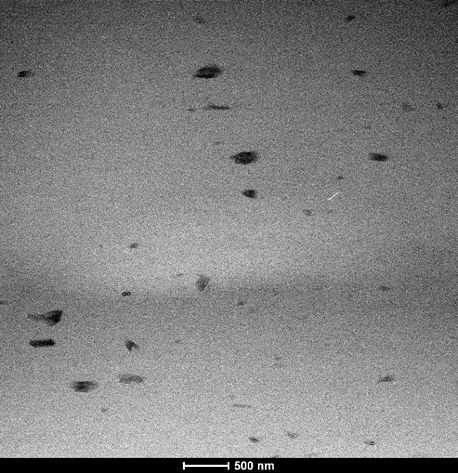 |
| --- | --- |
| (**a**) | (**b**) |
| 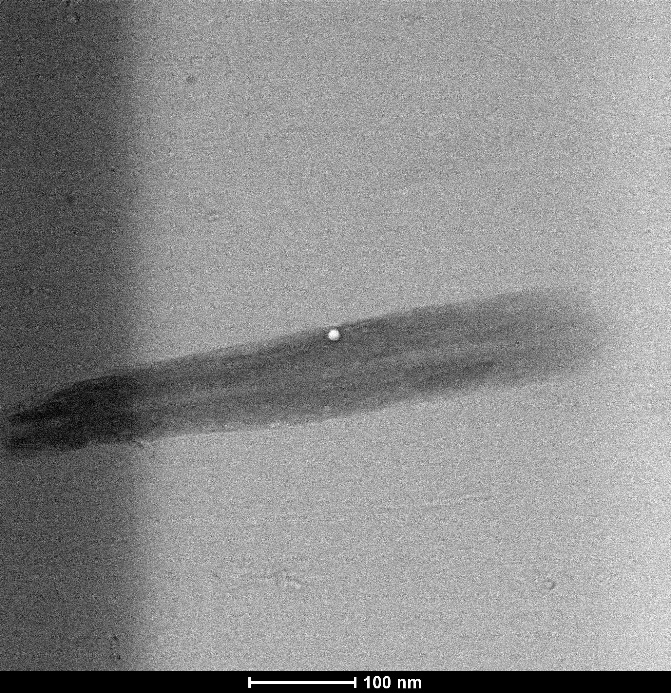 | 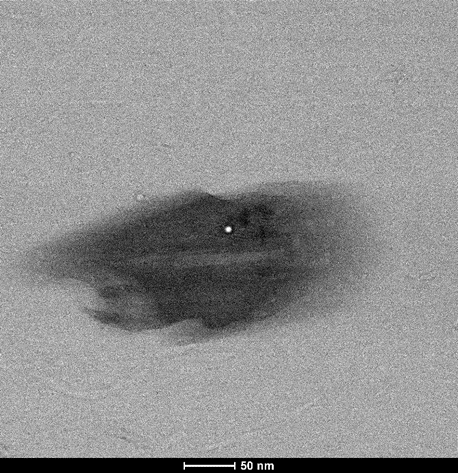 |
| (**c**) | (**d**) |
| 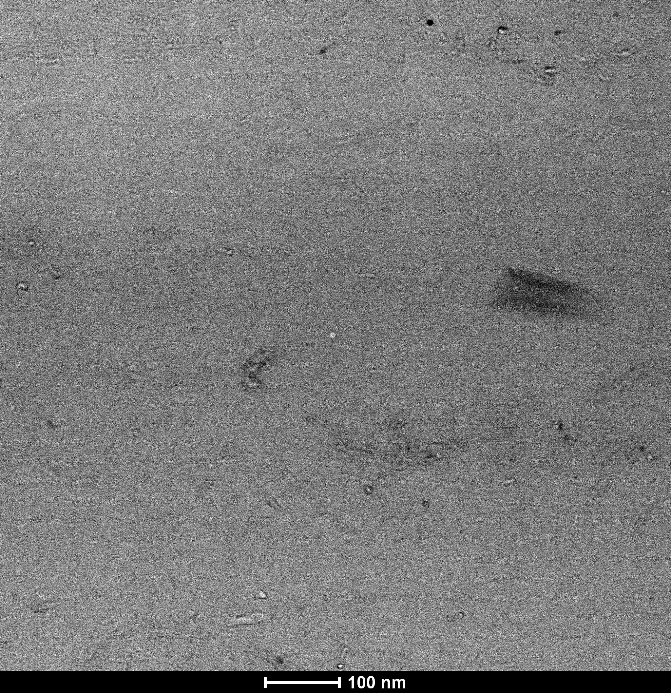 | 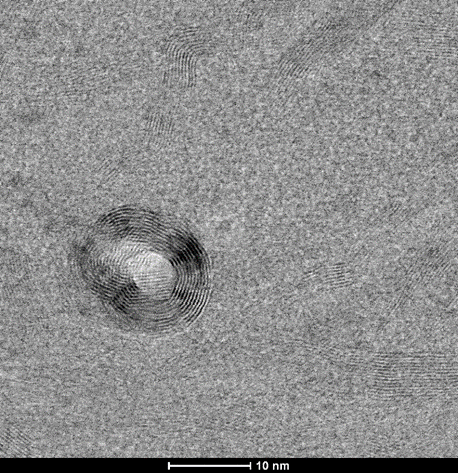 |
| (**e**) | (**f**) |
| 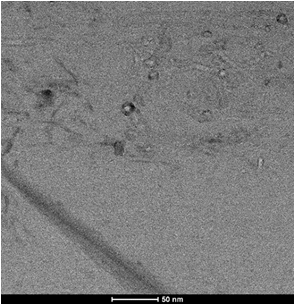 | 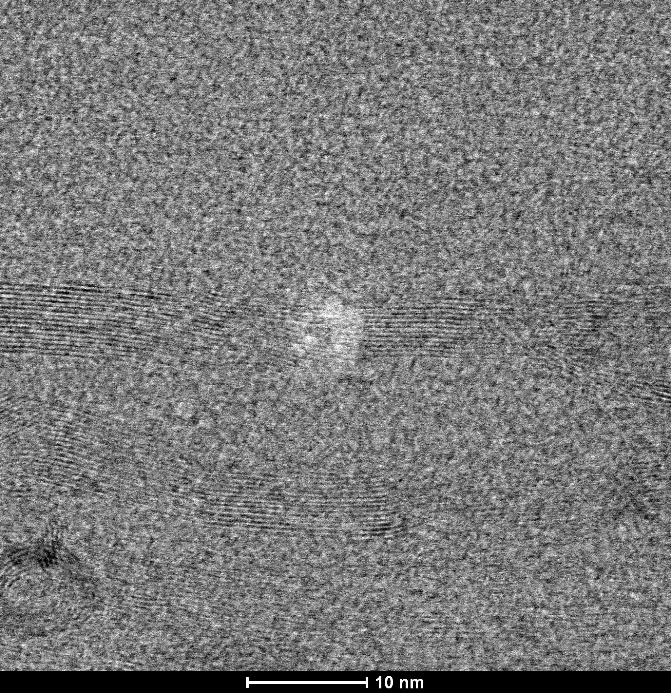 |
| (**g**) | (**h**) |

**Figure S1.** TEM images of EVA/6 wt.% CNT + HNT nanocomposite at different magnifications. Scale bars: 1 μm (**a**), 500 nm (**b**), 100 nm (**c**), 50 nm (**d**), 100 nm (**e**), 10 nm (**f**), 50 nm (**g**), 10 nm (**h**).

| 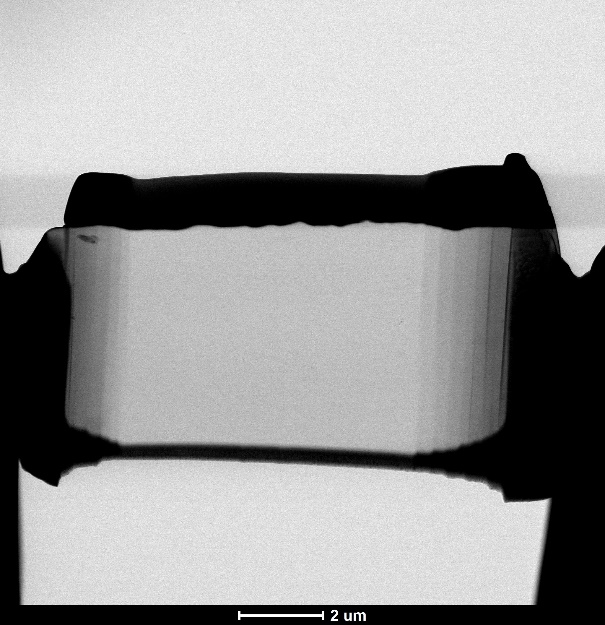 | 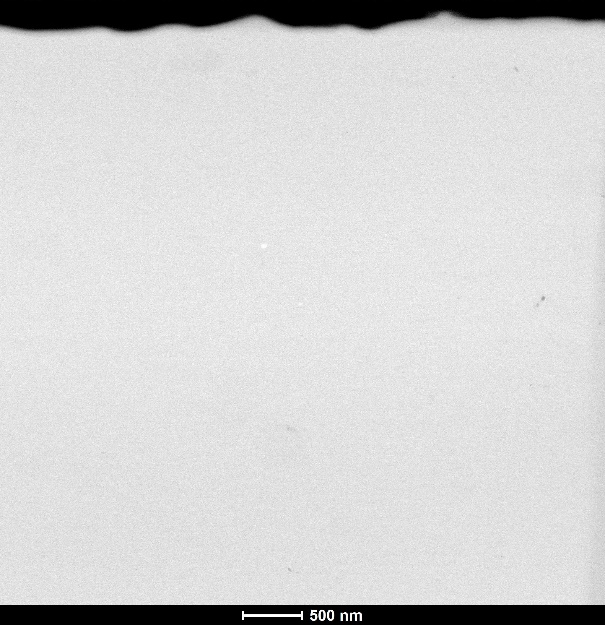 |
| --- | --- |
| (**a**) | (**b**) |
| 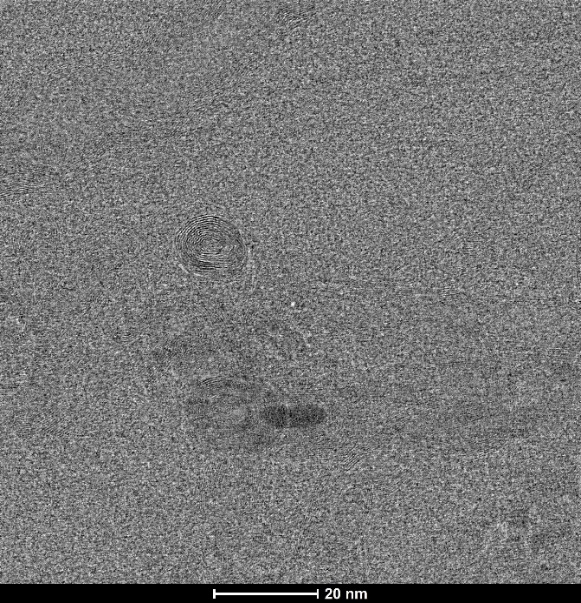 | 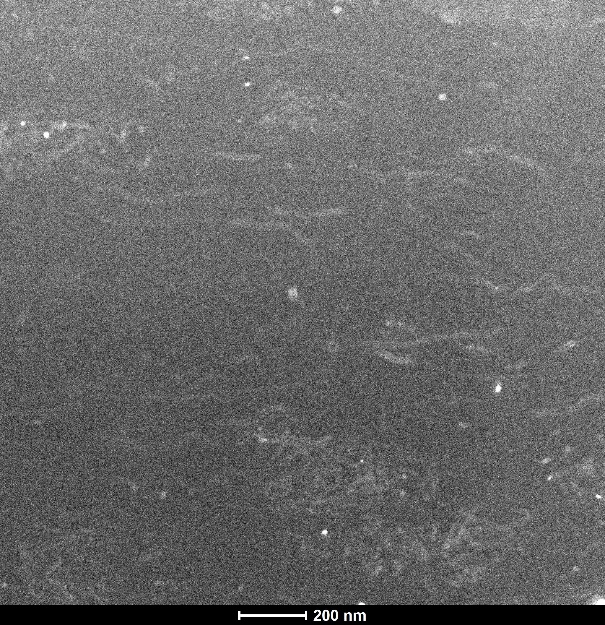 |
| (**c**) | (**d**) |
| 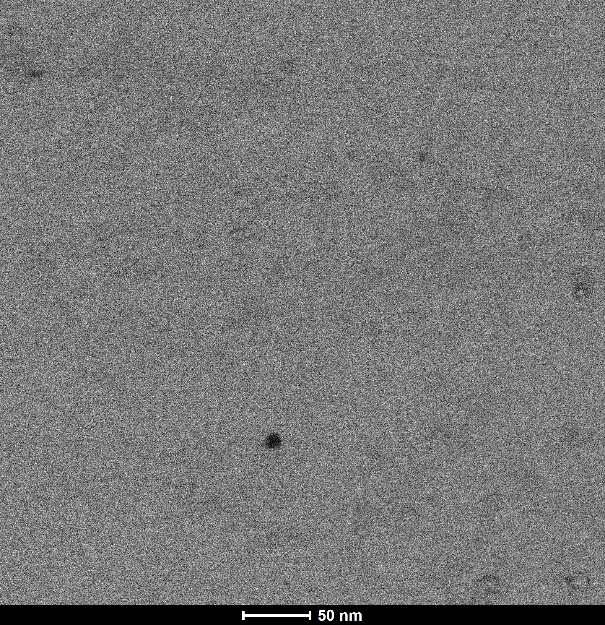 | 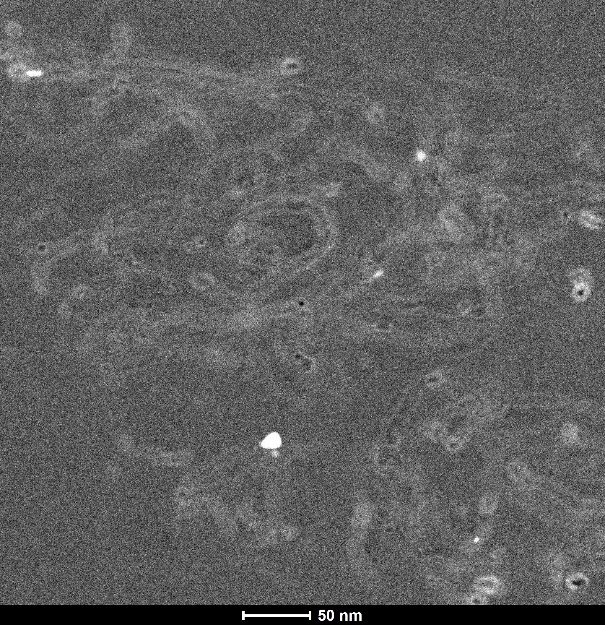 |
| (**e**) | (**f**) |

**Figure S2**. TEM images of EVA/6 wt.% CNTs nanocomposite at different magnifications. Scale bars: 2 μm (**a**), 500 nm (**b**), 20 nm (**c**), 200 nm (**d**), 50 nm (**e**, **f**).
